# Machine-assisted cultivation and analysis of biofilms

**DOI:** 10.1038/s41598-019-45414-6

**Published:** 2019-06-20

**Authors:** Silla H. Hansen, Tobias Kabbeck, Carsten P. Radtke, Susanne Krause, Eva Krolitzki, Theo Peschke, Jannis Gasmi, Kersten S. Rabe, Michael Wagner, Harald Horn, Jürgen Hubbuch, Johannes Gescher, Christof M. Niemeyer

**Affiliations:** 1 Karlsruhe Institute of Technology (KIT), Institute for Biological Interfaces (IBG-1), Herrmann-von-Helmholtz Platz 1, 76344 Eggenstein-Leopoldshafen, Germany; 2 Karlsruhe Institute of Technology (KIT), Institute for Applied Biosciences (IAB), Fritz-Haber-Weg 2, 76131 Karlsruhe, Germany; 3 Karlsruhe Institute of Technology (KIT), Institute of Engineering in Life Sciences, Section IV: Biomolecular Separation Engineering, Fritz-Haber-Weg 2, 76131 Karlsruhe, Germany; 4 Karlsruhe Institute of Technology (KIT), Engler-Bunte-Institut, Water Chemistry and Water Technology, Engler-Bunte-Ring 9a, 76131 Karlsruhe, Germany

**Keywords:** Biofilms, Microbial communities

## Abstract

Biofilms are the natural form of life of the majority of microorganisms. These multispecies consortia are intensively studied not only for their effects on health and environment but also because they have an enormous potential as tools for biotechnological processes. Further exploration and exploitation of these complex systems will benefit from technical solutions that enable integrated, machine-assisted cultivation and analysis. We here introduce a microfluidic platform, where readily available microfluidic chips are connected by automated liquid handling with analysis instrumentation, such as fluorescence detection, microscopy, chromatography and optical coherence tomography. The system is operable under oxic and anoxic conditions, allowing for different gases and nutrients as feeding sources and it offers high spatiotemporal resolution in the analysis of metabolites and biofilm composition. We demonstrate the platform’s performance by monitoring the productivity of biofilms as well as the spatial organization of two bacterial species in a co-culture, which is driven by chemical gradients along the microfluidic channel.

## Introduction

Biofilms are the most widespread form of microbial life in all kinds of partly extreme habitats on earth. They consist of a variety of different cell types embedded in an extracellular matrix and form complex three-dimensional structures. These ubiquitous communities play a major role for human health and our environment and might even serve as tools for the next generation of biotechnological processes^1–3^. However, due to their enormous complexity and dynamics, biofilms are very difficult to study, manipulate, model and control for specific applications. In general, improved control and handling of complex systems can often be achieved by the use of machine-assisted methods which provide well-defined experimental conditions and minimise variations caused by errors due to manual handling. In fact, this strategy is currently being used in basic and applied research in chemistry and biomedicine to make more efficient use of human resources and improve the safety, efficacy, and scope of development and production processes. For example, the implementation of machine-assisted programs that build on solid-phase syntheses aided by fluid handling has already led to impressive advances in synthetic organic chemistry^4–7^ and the manufacturing of pharmaceutical compounds^8,9^. These developments are complemented by current advances in the design of microfluidic lab-on-a-chip systems for applications in the life sciences, which include fundamental studies of cellular processes, biomedical diagnostics and drug discovery as well as biocatalysis^10–14^.

While few studies already illustrate the advantage of automated methodologies for biofilm research^15–17^, several studies have applied microfluidic techniques to the field of biofilm analysis, primarily to investigate biofilm formation of model organisms that are often genetically modified in order to be traceable^17–22^. Most of these studies use singular approaches like optical microscopy or microbiological methods for the characterization of the cultured biofilms^23^. However, further progress in understanding biofilm behaviour will depend on robust, automated solutions for multivariate analysis of mixed species biofilms to elucidate basic principles that occur in these complex life forms or to exploit them as novel and superior biocatalysts for biotechnological processes^1,3,24^. As a step towards this goal, we here report on the development of an integrated platform for machine-assisted biofilm research. The focus was on the development of a generic, automatable approach that makes it possible to investigate native multi-species biofilms that are not accessible with genetic engineering methods. To this end, a number of modules have been developed combining powerful standard methods such as flow cell technology and optical imaging with new robotic approaches for spatiotemporal characterisation to enable investigation of variations in the composition of the biofilm community and the culture broth. In order to minimize the effort and possible errors caused by manual handling, we aimed at a high degree of automation for the entire biofilm platform.

**Figure 1:**
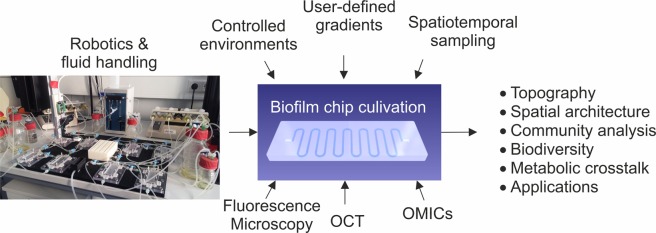
Overview of the integrated platform for machine-assisted cultivation and analysis of biofilms.

The system is based on microfluidic polydimethylsiloxane (PDMS) chips mounted on tailored interfaces to connect with hardware for automated liquid handling and instrumental analysis, such as fluorescence reading, epifluorescence microscopy, optical coherence tomography (OCT) and liquid chromatography (Fig. 1). The platform is operable under oxic and anoxic conditions, allowing for different gases and nutrients as feeding sources and offers high spatiotemporal resolution in the analysis of metabolites and biofilm composition. We demonstrate the platform’s applicability and versatility by investigation of the self-organized separation of mixed cultures along autonomously created chemical gradients in flow chips. Furthermore, the system was used for the characterization of a functional biofilm that acts as a whole cell biocatalyst in the stereoselective reduction of a prochiral diketone substrate. We believe that the here presented implementation of machine-assisted microfluidics, robotic handling, and in-depth instrumental analysis is an important advance for the exploration and exploitation of biofilm development and community dynamics.

## Results

### The automated platform for biofilm cultivation and analysis

As illustrated in Fig. 1, our platform is based on microfluidic channel structures, which can be readily produced from the elastomer polydimethylsiloxane (PDMS) by replica casting using micromolds prepared by micromachining or photolithographic techniques^25^. While in principle various different materials like for example glass, duroplastic or thermoplastic materials, polymethylmethacrylate (PMMA) and thiol-enes can be used for the manufacturing of the flow cells^26^, we have used the elastomer PDMS because it offers many decisive advantages. It is highly biocompatible, so that it can be used routinely as a standard material in biomedicine, e.g. for implants, in addition to the cultivation of prokaryotic and eukaryotic cells^27^. It is very easy to process into any shape by replica casting. Furthermore, after processing, it retains its elastic properties, which allows to puncture the material for sampling purposes without causing irreparable damage in the form of holes and leaks. Of particular importance for cell culture applications is that PDMS has a very high gas permeability, which allows the fluidic system to operate under well defined environmental conditions in terms of gas composition (see below).

The platform’s flow-chip modules are designed to provide full connectivity to peripherals independently of the size and shape of the embedded channel geometries. Therefore, any channel geometry with different heights and widths can be integrated into the platform, e.g. to generate thick, multi-layer biofilms in very deep channels or to generate turbulent flow profiles using special microfluidic geometries^28^ to simulate the conditions of ‘real world’ medical and environmental settings, such as those occurring in blood flows, water pipes or heat exchangers.

The PDMS chips are sealed with coverslips to enable the accurate microscopic inspection of the microchannel’s interior. The fluidic chips are mounted in cartridges, which serve as the interface to pumps and valves for media supply to enable cultivation of biofilms under controlled conditions for prolonged periods. Depending on the species and the selected experimental conditions, microbial communities form thick biofilm layers which tend to clog the cultivation channels. Possible measures to prevent *clogging* in flowcell experiments include the increase of flow rates (and thus shear forces), the reduction of the amount of nutrient supplied and/or the shortening of the cultivation time. In our experiments we found that linear channels with dimensions in micrometer range (200 µm) tend to clog very quickly, whereas this problem was significantly reduced in the here used meander channel (1 × 0.5 mm²) (Fig. S1b). Indeed, this setting allowed us to conduct cultivation campaigns of up to more than 12 months (data not shown). Non-destructive *in situ* imaging of living biofilms was performed by means of light microscopy (incl. epifluorescence) and optical coherence tomography (OCT)^29^. Since PDMS is gas permeable, a casket was developed, which allows long-term anoxic cultivation as well as the use of arbitrary gas mixtures as carbon and electron sources (Supplementary Fig. S1h). Furthermore, to enable online measurement of oxygen inside the microfluidic flow cells, methods for the integration of fiber-optical sensors were developed (Supplementary Figs S1, S2). A cartridge was developed which functions as interface to a liquid handling station (LHS, Supplementary Figs S3, S4), a widely established automation tool in biological laboratories. Here, the LHS was utilized to enable automated end-point analysis of the biofilms by fluorescence *in situ* hybridization (FISH) or catalyzed reporter deposition FISH (CARD-FISH), as discussed below. Importantly, the standardized technical interface allows to follow the automated FISH procedures by imaging techniques like OCT, for instance, to quantify mechanical abrasion during the various washing and incubation steps of the FISH procedure (Fig. S5).

**Figure 2:**
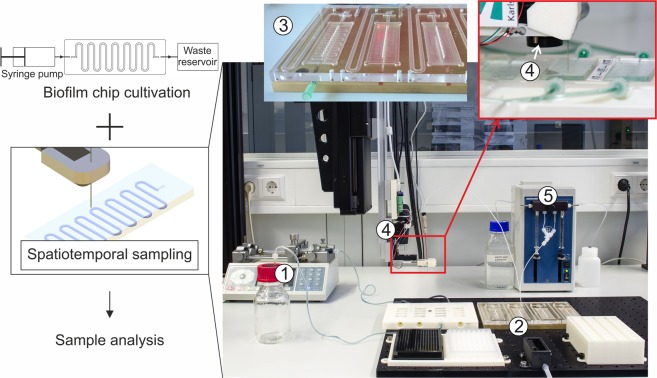
Robotic sampler for non-destructive spatiotemporal *in situ* analysis of flow cell-cultivated biofilms. The overview image shows external syringe pumps (1) used for continuous perfusion of the microfluidic bioreactors with medium or substrate, the robotic deck of the sampling device (2) onto which a custom-made temperature-controlled chip holder is mounted (3), the sampling head (4) that is connected to the pumping unit (5) for withdrawal of small sample volumes from the channel. Further details on the design of hardware parts and control software are shown in Supplementary Figs S6–S10.

The central instrument of the developed platform is a robotic sampler that allows to repeatedly draw liquid sample volumes directly from arbitrary positions of the microfluidic channel without interruption of a running cultivation (Fig. 2, see also Supplementary Figs S6–S10). The sampler is equipped with a sharp cannula, connected to pumps for sample extraction, which is freely movable in either X-, Y- or Z-direction (600 mm × 300 mm × 200 mm, respectively) with a precision of ±25 μm in X- and ±10 μm in Y- and Z-direction. Exact positioning of the cannula is controlled by an automated pattern recognition software (Supplementary Fig. S7) to assure precise repeated sample drawing over the entire experiment. The vertical positioning of the needle is controlled by a pressure sensor (Supplementary Fig. S8) to enable the puncturing of the PDMS layer on top of the channel, withdrawal of either liquid from above or cell material from within the biofilm, and subsequent delivery of the samples to microplates. After sampling, the flexible PDMS layer seals back to leave a closed channel that enables continued cultivation. The collected samples are transferred to a cooled storage compartment (Fig. S9) from which they can be subjected to further analysis by e.g., chromatography or sequencing (discussed below). The robotic sampler is controlled by a graphical user interface that uses prescribed script modules to conduct the various procedures necessary for automated sample extraction from the microfluidic chips (Fig. S10).

### Implementation of automated FISH procedures for endpoint analysis of flowcell cultivated biofilms

**Figure 3:**
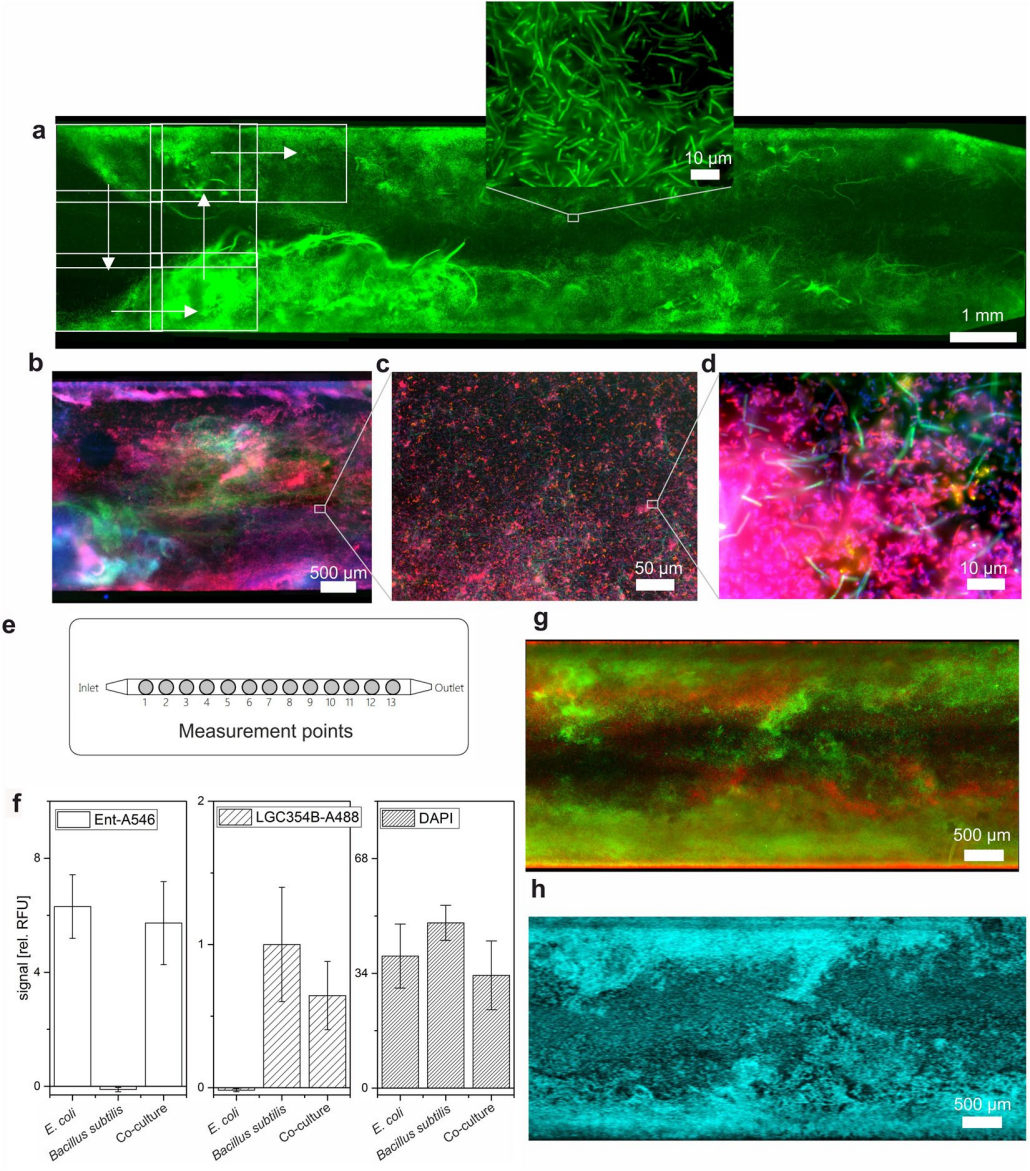
Optical analyses of flowcell biofilms labeled by automated FISH. Overview image and high-resolution (inset) micrographs of a pure *B*. *subtilis* (**a**) and a mixed species *E*. *coli* and *B*. *subtilis* biofilm of (**b**–**d**). Biofilms were grown in triplicates in linear flow chambers for 12 h (**e**), subjected to automated FISH and analyzed with the integrated fluorescence reader at the 13 measurement points to obtain mean fluorescence values for the nine parallelly cultivated biofilms (**f**). *B*. *subtilis* and *E*. *coli* were labeled with probes LGC354B-A488 (colored in green) and Ent-A546 (red), respectively. Error bars represent the standard deviation between the mean fluorescence values of triplicate experimental samples carried out in individual flow chips. Note that DAPI staining indicates approximately equal growth density and FISH data allow for reliable identification of pure *E*. *coli* and *B*. *subtilis* cultures. (**g**) Epifluorescence micrographs of mixed species *B*. *subtilis* and *E*. *coli* biofilms (same color code as in **a**–**d**) confirm the topographical biofilm features visualized by means of OCT (**h**). Displayed height = 100 μm (cyan colored).

FISH or CARD-FISH are the gold standard in biofilm research because they allow for the analysis of the composition of microbial communities with an extraordinarily high spatial resolution, multiplexing capability and sensitivity^30,31^. A disadvantage of this technique is that the experimental execution of FISH requires multiple, time consuming steps for labeling of the ribosomal RNA (rRNA) targets with fluorescent oligonucleotide probes. To overcome this obstacle and motivated by approaches to the semi-automated conduction of FISH for eukaryotic cell culture^32–34^, we developed a technical interfaces that enabled implementation of automated FISH/CARD-FISH analyses into our robotic platform. Based on a previous design^35^, the dimensions, contact points and connections of our flowcell-to-LHS interface (Supplementary Fig. S4) are compatible with standard microtiter plate equipment, such as thermocyclers, shakers and plate readers. Hence, the automated system conducts all steps of the FISH procedure autonomously. To demonstrate the system’s utility, we carried out fully automated FISH analyses of single- and mixed-species biofilms of the gram-negative organism *Escherichia coli* and the gram-positive strain *Bacillus subtilis* that were grown in triplicates in linear flow chips with constant medium supply (Fig. 3). See also Supplementary Information for a detailed description of the experimental procedure (Figs S3 & S4).

With this setup, it was possible to characterize the biofilms with the LHS-integrated fluorescence reader (see Supplementary Fig. S3) as well as to image them by epifluorescence microscopy. The analysis of predefined spots inside the linear channels with the fluorescence reader allowed to clearly distinguish between single and mixed species biofilms (Fig. 3e,f). However, owing to the limited lateral resolution of conventional plate readers, the accurate and quantitative mapping of biofilm composition required high-resolution fluorescence imaging. To this end, sequential images were taken (indicated by rectangles, in Fig. 3a) and merged afterwards by grid stitching^36^ to document with high resolution large areas of up 3 × 14 mm inside the flowcell. The method allows to reveal mesoscopic features, such as the streamers formed by the *B*. *subtilis* inside the broader context of the flowcell (Fig. 3a), as well as the microscopic composition of mixed species biofilms (Fig. 3b–d). Like all FISH protocols the method relies on the fixation of the cultured cells (Fig. S3) and therefore is only suitable for endpoint analysis of the biofilms. The results clearly illustrate the advantages of the developed method for multiscale FISH imaging of native biofilms directly inside their cultivation vessel. Furthermore, comparison of OCT images with the final FISH images revealed that biofilm features visualized by OCT are easily recognizable by epifluorescence microscopy after FISH (Fig. 3g,h). The implementation of OCT also clarified that only slight biofilm detachment occurred during the automated FISH procedure and that the overall structural integrity was preserved (Fig. S5).

### Interdependent two-species biofilms

As a result of microbial activity and/or the diffusion of gases out of or into the medium via the PDMS materials, the microfluidic channels of our platform allow for the formation of gradients along the medium flow. This feature can be exploited for the cultivation of interdependent communities that are fluidically connected but spatially separated. The self-organized separation of mixed cultures along autonomously created gradients can be controlled and steered by the applied growth conditions and it may also allow to determine specific characteristics of novel environmental isolates, such as growth auxotrophies that are difficult if not impossible to study by conventional approaches.

As a proof of concept, we studied the interaction of a chromate resistant *Leucobacter chromiiresistens* strain with the laboratory model organism *E*. *coli*. *L*. *chromiiresistens* has the ability to tolerate the highest chromate concentrations reported so far^37–39^. It is an aerobic organism whose chromate tolerance stems from its capability to efficiently reduce toxic Cr(VI) to Cr(III) species, the latter of which are highly insoluble and therefore less toxic. Due to the detoxifying properties of *L*. *chromiiresistens*, our hypothesis was that flow cultivation of mixed *E*. *coli/L*. *chromiiresistens* cultures with Cr(VI)-containing medium should lead to a spatial separation of the two species. The front part of the channel should be inhabited mainly by *L*. *chromiiresistens*, while *E*. *coli* should accumulate in the lower parts of the chip. To achieve an optimal characterization of the biofilm composition, we took advantage of both the automated FISH procedure and the robotic sampler. The latter was used to draw 10 μl samples from the biofilms, which were subjected directly to 16S rRNA gene amplification and subsequent Illumina sequencing.

**Figure 4:**
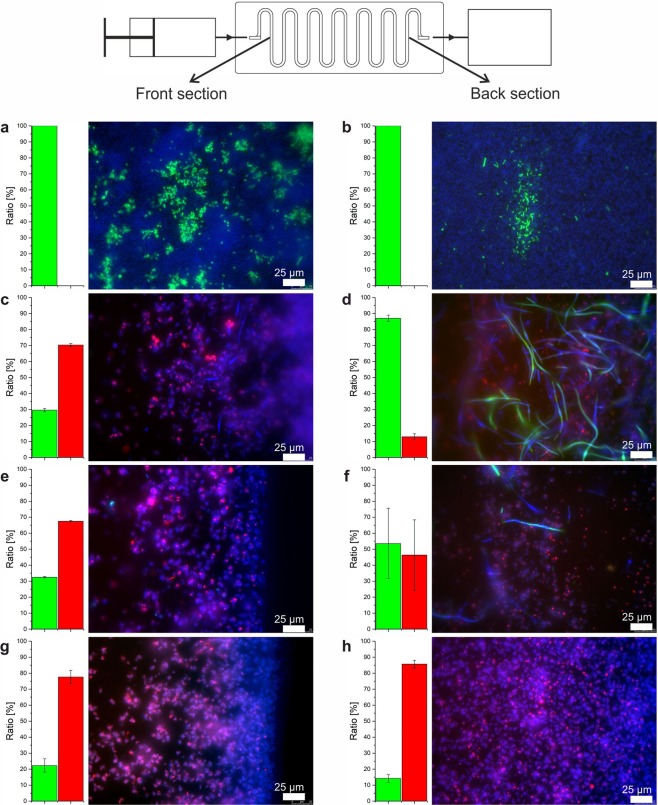
Interdependent biofilm consortia self-organize along an autonomously created gradient of chromate. A series of four identical inocula was used and cultivated for seven days in the absence (**a**,**b**), or the presence of 1 mM (**c**,**d**), 2 mM (**e**,**f**) or 3 mM (**g**,**h**) chromate containing medium. The analysis was performed by robotic cell sampling and subsequent staining by the auto FISH procedure. Epifluorescent images and phylogenetic composition in (**a**,**c**,**e**,**g**) represent the front section of the microfluidic chip while (**b**,**d**,**f**,**h**) show representative data from the rear end. The error bars represent the standard deviation between the ratio of reads from the 16S rDNA amplicon sequencing of two independently cultivated flowcells. Sampling points are indicated in the scheme of the setup. *E*. *coli* cells are shown in green, *L*. *chromiiresistens* cells in red. Unspecific labeling with DAPI is indicated in blue. The bar charts on the left side of each sample represent the community composition derived from 16S rDNA amplicon sequencing.

We conducted parallel co-cultivation experiments for seven days in LB-medium that contained variable chromate concentrations ranging from 0–3 mM. In the absence of chromate, the biofilm consisted almost exclusively of *E*. *coli* cells throughout the entire flow channel (Fig. 4a,b). Hence, in the absence of toxic chromate, the higher growth rates of *E*. *coli* led to overgrowth of *L*. *chromiiresistens* species. In the presence of 1 mM chromate, formation of two separated growth areas was observed with an abundance of *L*. *chromiiresistens* and *E*. *coli* species at the front and rear regions of the channel, respectively (Fig. 4c,d). While *L*. *chromiiresistens* dominated the oxic part containing the highest chromate concentration, *E*. *coli* could thrive in the rear parts of the channel presumably due to the decrease in Cr(VI) concentration and its ability to thrive as facultative anaerobic organism. Of note, the elongated cell shape of the *E*. *coli* cells is a known consequence of chromate stress^40^. A further increase of the chromate concentration led to biofilms that were dominated by *L*. *chromiiresistens* cells throughout the channel (Fig. 4e–h). However, in the case of 2 mM Cr(VI) concentration, 16S rDNA amplicon sequencing still clearly revealed preferred *E*. *coli* growth at the rear end of the channels (Fig. 4f). These data indicate that the amount of *L*. *chromiiresistens* biomass grown in the channels’ front part has a sufficient capacity to reduce about 1 mM Cr(VI) while, at higher concentrations, the remaining chromate could be no longer efficiently removed to allow growth of *E*. *coli*. Hence, the results provide a clear demonstration that interdependent consortia self-organize along an autonomously created chemical gradient into spatially separated populations.

### Productive biofilms

**Figure 5:**
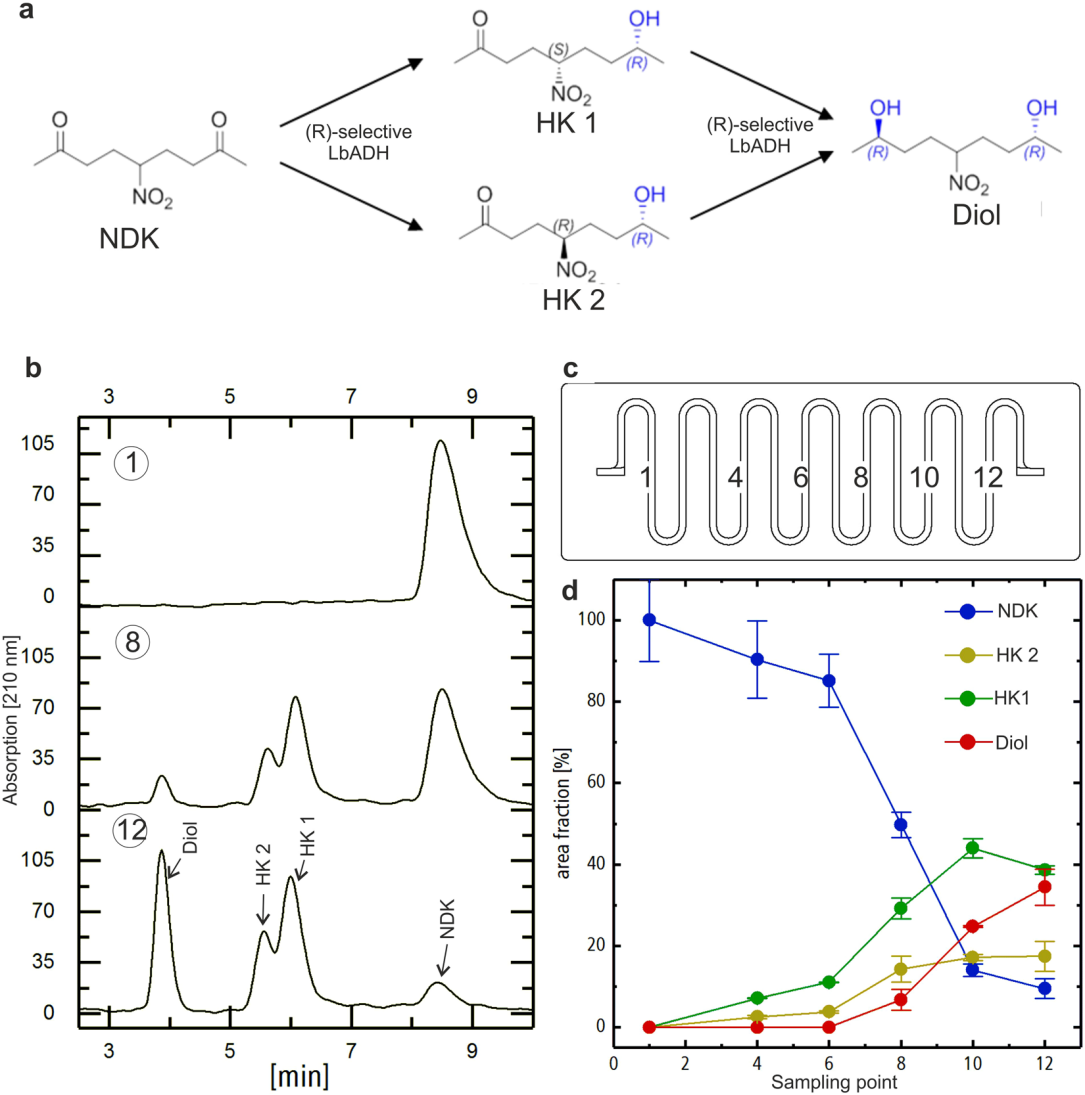
Culturing and analysis of a productive *E*. *coli* biofilm. (**a**) Reaction scheme of the stereoselective reduction of the prochiral nitro-diketone substrate (NDK) into hydroxyketone (HK) and diol products by the *R*-selective ketoreductase LbADH, expressed in a fluidically-cultivated *E*. *coli* biofilm cultivated for 42 h. (**b**) Representative chiral HPLC chromatograms of samples drawn from selected points of the meandric cultivation channel (**c**). The decrease of NDK educt and HK/diol products is clearly evident from the analysis of the reaction samples retrieved along the flowpath at a constant flowrate of 2 µL/min (**d**). The error bars represent the standard deviation of two independent samples, which where withdrawn sequentially from the indicated sampling points.

Applied biofilm research is concerned with the exploitation of bacterial communities containing novel and superior biocatalysts for biotechnological processes^3^. Hence, to illustrate the scope and utility of our platform, we performed microfluidic cultivation experiments with a productive *E*. *coli* biofilm that expresses the *R*-selective alcohol dehydrogenase LbADH from *Lactobacillus brevis* ATCC 14869. As a test case for the continuous, spatially resolved monitoring of the catalytic activity of the cultured biofilm, we used the sequential biocatalytic reduction of 5-nitrononane-2,8-dione (NDK, in Fig. 5a) by LbADH that leads to selective production of the hydroxyketones HK1 and HK2, which are then converted into the pseudo-C2-symmetric *R*,*R*-configured diol product^41,42^. Using the robotic sampler, samples were taken from 12 different points along the meandric cultivation channel. Analysis by chiral HPLC clearly revealed that the NDK educt depleted while the HK intermediates and diol product emerged with increasing path length. (Fig. 5b–d). Together with the results from the metabolic analysis of interdependent biofilms described above, this data clearly illustrates the suitability of our platform for multivariate analysis of complex biofilms.

## Discussion

The developed platform for machine-assisted cultivation and analysis of biofilms is ideally suited to advance basic and fundamental research on synthetic as well as natural multispecies biofilms. Cultivation can be pursued under arbitrary environmental temperature and gas phase conditions to mimic a large variety of natural habitats. Importantly, our system allows for culturing under flow conditions to establish shear forces that are an essential factor for controlling growth in native environments. The standardized dimensions of the flowcell chips allow for the facile integration of a large variety of designs that are readily accessible by rapid prototyping and soft lithography and can be conveniently adjusted in their length, curvature and internal channel dimensions. This enables diverse applications ranging from rapid growth experiments, as exemplified here with binary *E*. *coli* and *B*. *subtilis* cocultures (Fig. 3), to long-term studies for investigation of the self-organized separation of mixed cultures along autonomously created gradients (Fig. 4) and the performance of productive biofilms (Fig. 5).

The here presented biofilm studies show the broad application spectrum of our biofilm analysis platform. Experiments were routinely run as experimental duplicates or triplicates on individual flow chips. The data obtained clearly indicate that flow culture experiments can be carried out in a reproducible manner so that internal biological processes can be disclosed. But also with our experimental platform, the results show a variance between the individual cultured biofilms, which indicates that multispecies biofilms are complex life forms that react sensitively to small fluctuations in their environment. One important aspect of ongoing work with our platform is therefore to reveal the exact possibilities and limits of reproducibility. Furthermore, since sequential sampling at identical local coordinates is possible (Fig. 5d), dynamic studies can also be performed to analyse temporal fluctuations on relevant time scales of biofilm growth.

A very important feature of the flow chips is their technical connectivity to commercial liquid handling systems and established analytical instrumentation for imaging. We used a combination of these opportunities to develop automated FISH- and CARD-FISH protocols that unburden the user from time consuming experimental steps and even allow for increased throughput by multiplexing chip-based cultivation experiments. OCT analysis was also implemented in the workflow for online monitoring of biofilm growth and to correlate the fluorescence images to the *in vivo* situation before fixation and labeling. The OCT controlled automatization increases the validity of the acquired images as the scientist is no longer a source for operational errors. While automated microscopy can conveniently be implemented for image analysis, it might not even be necessary in certain applications because a plate reader output was shown to reliably help to distinguish between different biofilm-populations. Of note, the migration of FISH-probes into denser parts of the biofilms might be diffusion limited and a depletion of the probes while being pumped from the beginning to the end of the chip might occur. Together with variations of cellular activity, these effects could be the reasons for the observed differences in staining efficiency of FISH- and DAPI in Fig. 4.

16S rRNA gene amplicon sequencing was used to study a synthetic two species biofilm of *Leucobacter chromiiresistens* and *E*. *coli*. Changing compositions of the biofilm along the flowpath as well as with varying feed medium could be observed. It was also confirmed by subsequent FISH analysis that *L. chromiiresistens* created a niche for *E*. *coli* cells in the rear part of the flowcell by the reduction of toxic chromate. Furthermore, it could be shown how the online sampling in combination with HPLC can be used to determine concentration levels of metabolic compounds and biotechnologically-relevant products. On the one hand, this approach gives access to the composition and dynamics of the microbial community cultivated within the flowcells and, on the other hand, it demonstrates the possibility to evaluate online process parameters for biocatalytic transformation reactions. The latter is considered one of the basic prerequisites for the breakthrough of the use of productive biofilms in biotechnological applications.

In summary, microfluidic biofilm cultivation devices and tailored robotic instrumentation were combined with powerful standard methods such as (CARD-) FISH, HPLC and next generation sequencing to create a unique set of tools for multispecies biofilm research. All here described techniques for biofilms analysis do not rely on the genetic labelling of cells and, therefore, enable the investigation of native communities. Emerging research fields, for example, studies on productive biofilms or the exploration of complex native consortia will largely benefit from the developed platform. It is therefore anticipated that this work represents an important step towards the realization of machine-assisted processes for the next generation of biotechnology.

## Methods

### Chip fabrication

The microfluidic chip designs were based on the dimension of standard microscope slides (76 × 26 mm² DIN ISO 8037-1:2003-05). The upper part containing the cultivation channel was manufactured by replica casting of polydimethylsiloxane (PDMS) (Sylgard 184, Dow Corning, USA) in brass replication molds. A glass cover slip (76 × 26 mm², thickness 170 µm) was bonded to the bottom of the PDMS chip via oxygen plasma treatment. The meandering channel had a rectangular cross section of 500 × 1000 µm² and a total volume of 150 µL (Fig. S1b). The straight channel for biofilm cultivation was 3 mm wide, 1 mm high and 54 mm long (Fig. S1a). The layout of the flowcell was designed in accordance with the spacing of a microwell plate, enabling optical analysis with standard microplate readers. The minimal spotsize for fluorescence measurements in the here used microplate reader (Infinite M200 pro, Tecan, Switzerland) was 3 mm corresponding to the channel width of the straight cultivation flowcell. Cannulas (Sterican, B. Braun Melsungen AG, Germany), were inserted through horizontal holes in the molds before pouring the PDMS prepolymer to serve as placeholders for the later connection channel. The PDMS was cured at 60 °C for at least 3 h. For connection to the silicone tubing (Tygon tubing R3603 (ID = 1.6 mm) Saint-Gobain, France) standard cannulas and luer lock fittings were used (Supplementary Fig. S1c, h).

### Robotic sampler

For x-y-z positioning, high precision linear stages with step motors (LIMES, Owis, Germany) were used. A dedicated motor controller (PS 90, owis, Germany) equipped with three control modules for the individual axes was used for operating the movements. The linear stages were mounted upside down on an optical portal in order to attain a robotic liquid handling arm. The probing cannula (OD = 400 µm) was mounted on a sensitive loadcell (4) (Böcker Systemelektronik, Germany) (Supplementary Fig. S8). A pumping unit (5) (MicroLab 500B, Hamilton, USA) with two independent syringe modules was installed for liquid handling. The first pump module equipped with a 25 µL glass syringe (ILS, Germany) enabled precise sampling in the microliter scale whereas the second module with a 1 mL glass syringe (ILS, Germany) allowed fast flushing of the whole system. Custom made 3D printed (DesignJet Color 3D, HP, USA) accessories were organized on a 400 × 600 mm² metric aluminium breadboard (spacing 25 mm, Thorlabs, USA). Thereby, a customizable layout with fixed positions of all components was realized (Supplementary Fig. S6a). The exact positions of the fluidic chips were determined automatically by a camera. Two reference points on the chip that were directly incorporated in the replication master were recognized by the image analysis tool (AForge.NET framework, circle detection, http://www.aforgenet.com/). This allowed for the calculation of the coordinates of predefined sampling points (Supplementary Fig. S7). The calibration of the camera to needle offset was done manually. The robotic sampler system was controlled by a custom-made software with a graphical user interface (Supplementary Fig. S10). Further technical details of the robotic setup and the control software will be published elsewhere.

### *B. subtilis* and *E. coli* biofilms

*E*. *coli* DH5α and *B*. *subtilis* DSM1088 were cultured in the straight flowcell using LB as feed medium. In one experiment triplicates of each biofilm where cultivated in parallel. Before inoculation the whole system was equilibrated in medium for at least 12 h. For the pure culture biofilms, the system was then inoculated with overnight cultures of both species, which were diluted to an optical density of 0.1 at 600 nm (OD_600_). For the two-species-biofilms, the overnight cultures were adjusted to an OD_600_ of 0.2 and pooled afterwards in a ratio of 1:1. After inoculation, the flow was halted for 1 h to allow for initial attachment of the cells. The biofilms were then cultivated at 37 °C for 12 h with a constant medium supply at 50 µL/min flowrate. In the FISH procedure, *B*. *subtilis* was visualized by hybridization with Alexa Fluor 488 labeled LGC354B probe^43^. *E*. *coli* was stained with Alexa Fluor 546 labeled ENT probe^44^.

### *L. chromiiresistens* and *E. coli* biofilms

Meandric chips were used to cultivate *Escherichia coli* K12 and *Leucobacter chromiiresistens*. Both organisms were pre-cultivated overnight at 30 °C with LB (lysogenic broth)-medium. An inoculum suspension with an OD_600_ of 0.15 per organism was pumped through the chips for 45 min with a flow rate of 1 µl/min. Thereafter, LB medium containing 0, 1 mM, 2 mM or 3 mM potassium chromate (K_2_Cr(VI)O_4_) was pumped through the chips with a flowrate of 2.5 µl/min. After seven days of cultivation, cell samples for 16S-sequencing were taken by the robotic sampling device right before biofilms were fixed by applying LB-medium containing 4% formaldehyde. The biofilms were analysed by automated FISH procedure with probes Ent (5′-CCC CCW CTT TGG TCT TGC-3′^45^ conjugated with Atto488) and HGC69a (5′-TAT AGT TAC GGC CGC CGT-3′^46^, conjugated with Alexa546). All chips were counterstained with DAPI. For 16S-sequencing analysis, templates consisted of 1 µl of the robotically withdrawn samples. Primers F_NXT_Bakt_341F (5′-CCT ACG GGN GGC WGC AG-3′) and R_NXT_Bakt_805R (5′-GAC TAC HVG GGT ATC TAA TCC-3′) were used for amplification. Sequencing was conducted by IMGM Laboratories GmbH (Martinsried, Germany) on an Illumina Miseq platform, using 2 × 250 bp paired-end (PE) reads. Signal processing, de-multiplexing, trimming of adapter sequences was conducted by IMGM Laboratories with the MiSeq Reporter 2.5.1.3 software. Further bioinformatic analysis of the 16S rRNA gene amplicon sequencing (primer cutting, quality and length trimming, merging, OTU clustering, and phylogenetic analysis) was carried out with the CLC Genomic Workbench software 10.0.1 equipped with the additional microbial genomic module 2.0.

### Catalytically active *E*. *coli* biofilms

The *E*. *coli* BL21 (DE3) was transformed with plasmid pET_LbADH-SBP^42^ and cultivated as biofilm in the meandering flowcell in selective LB medium for 24 h at 37 °C and 10 µL/min. The temperature was then reduced to 30 °C and the protein production was induced by addition of 0.5 mM ITPG to the cultivation medium for another 18 h. To evaluate the catalytic activity, the biofilm populated flowcells were placed on the deck of the robotic sampler, syringe pumps with media containing the substrate NDK (10 mM) were connected via polytetrafluorethylen (PTFE) tubing and biotransformation was carried out at 2 μL/min perfusion flowrate. After 1.5 h of equilibration two series of 10 μL samples were retrieved sequentially from various sampling points along the flowpath with the sampling robot and analyzed independently by a chiral HPLC method^41^.

## Supplementary information

BiofilmRobotics_SI

## Data Availability

The datasets generated during and/or analysed during the current study are available from the corresponding author on reasonable request.
