# Supplementary material for: Machine-assisted cultivation and analysis of biofilms
Source: Sci Rep. 2019 Jun 20;9:8933. doi: 10.1038/s41598-019-45414-6 (PMC6586868; doi:10.1038/s41598-019-45414-6)
Supplement: Supplementary file 1 — BiofilmRobotics_SI [file 41598_2019_45414_MOESM1_ESM.pdf]

Machine-assisted cultivation and analysis of biofilms

Silla H. Hansen[1], Tobias Kabbeck[2], Carsten P. Radtke[3], Susanne Krause[2], Eva Krolitzki[3], Theo Peschke[1], Jannis Gasmi[1], Kersten S. Rabe[1], Michael Wagner[4], Harald Horn[4], Jürgen Hubbuch[3], Johannes Gescher[1,2#], Christof M. Niemeyer[1,#]

Supplementary information

Content

Supplementary Figure S1:	Details of microfluidic flow cells and technical equipment for long-term biofilm cultivation.
Supplementary Figure S2:	Oxygen monitoring in abiotic and biotic experiments.
Supplementary Figure S3:	Hardware for robotic end-point analysis of flow cell-cultivated biofilms.
Supplementary Figure S4	Detailed description of the chip-to-LHS interface.
Supplementary Figure S5:	OCT analysis of biofilms during FISH procedure.
Supplementary Figure S6:	Robotic deck of the sampling device.
Supplementary Figure S7:	Automatic chip recognition.
Supplementary Figure S8:	Mounting of the probing cannula on a pressure-sensitive load cell.
Supplementary Figure S9:	Image series of the probing cannula entering the cultivation channel.
Supplementary Figure S10:	Graphical user interface for the control of the biofilm sampler.

[1] Institute for Biological Interfaces (IBG-1), Karlsruhe Institute of Technology (KIT), Herrmann-von-Helmholtz Platz 1, D-76344 Eggenstein-Leopoldshafen, Germany. [2] Institute for Applied Biosciences, [3] Institute of Engineering in Life Sciences, Section IV: Biomolecular Separation Engineering (BLT-MAB), Fritz-Haber-Weg 2, D-76131 Karlsruhe. [4] Engler-Bunte-Institute for Water Chemistry and Water Technology, Engler-Bunte-Ring 5, D-76131 Karlsruhe, Germany. Correspondence and requests for materials should be addressed to J.G. (email: johannes.gescher@kit.edu) or C.N. (email: niemeyer@kit.edu).

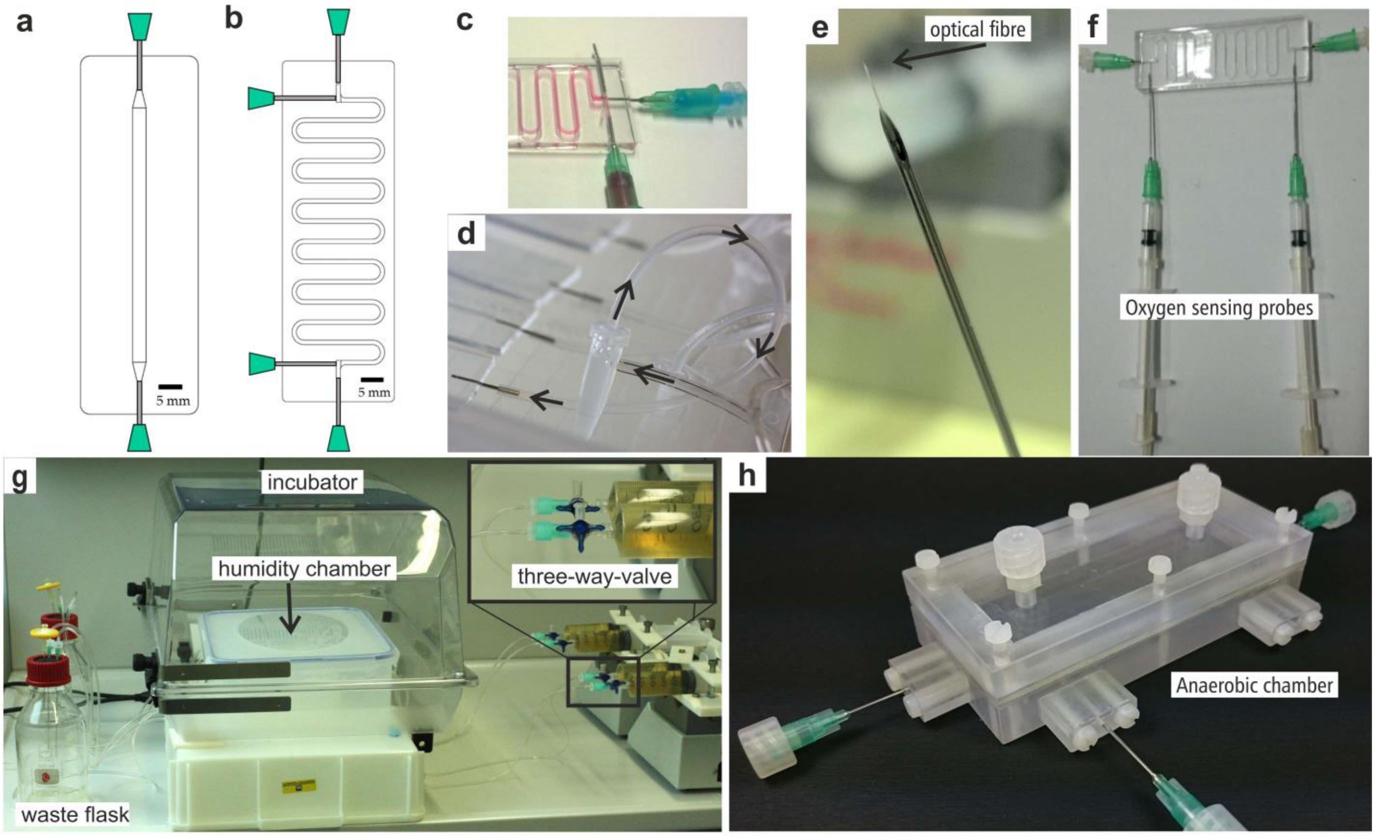

Supplementary Figure S1: Details of microfluidic flow cells and technical equipment for long-term biofilm cultivation. (a,b) Two different designs of PDMS chips were used in this work. Both layouts are based on standard dimensions of microscope slides to warrant compatibility with common microscopes. (c) PDMS chips are connected by sterile 0.8 mm cannulas to realize the leak free but reversible connection of the cultivation channels with standard syringe pumps. (d) Custom-made bubble-traps remove air bubbles from the feed medium. (e, f) Optical fibre micro sensors for oxygen measurement are moveably mounted within the cannula. (h) Representative image of a cultivation gasket for anoxic biofilm cultivation with a controlled gaseous phase and online oxygen measurement inside the microfluidic chip that is enclosed in the anaerobic chamber. (g) Overview image of a typical setup for long-term cultivation. A multi-channel syringe pump, equipped with large syringes, is used for constant medium supply. The chips are connected to the syringe through three-way-valves, to enable exchange of syringes during prolonged cultivation times. The chip is placed in a temperature-controlled humidity chamber to warrant constant environmental conditions. The effluent is collected in a waste flask.

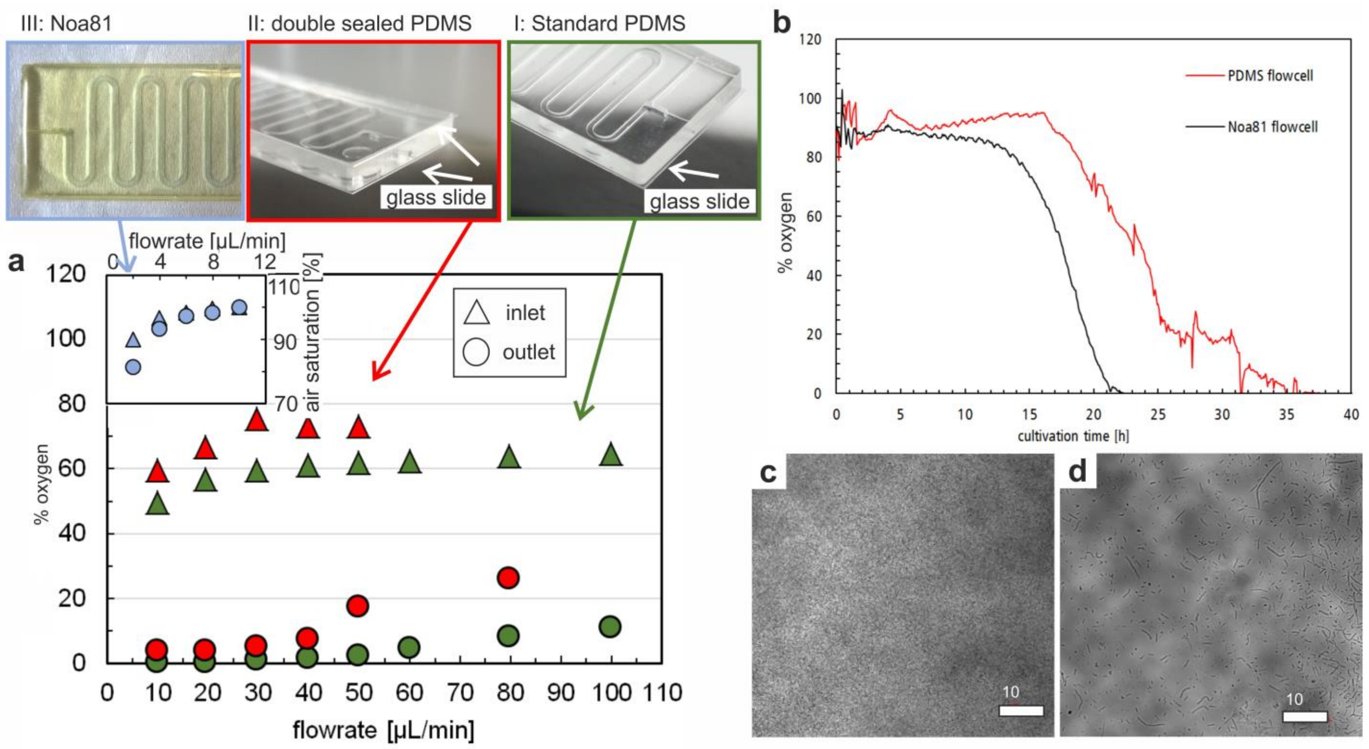

Supplementary Figure S2: Oxygen monitoring in abiotic and biotic experiments. (a) Three different meander flowcells, a standard PDMS cell on a glass slide (I, green symbols), a PDMS cell covered on both sides with bonded glass slides (II, red symbols), and a flowcell fabricated from the low gas permeable thiolen polymer Noa81 [1] (III, blue symbols) are compared. Oxygen concentration in dependency of the flowrate is determined at two positions of the flow channel, either at the inlet (triangles) or the outlet (circles), using optical fiber micro sensors shown in Figure S1D. The entire chip was held under anoxic conditions using nitrogen gas and flushed with air-saturated LB medium. In the standard PDMS chip (green), significant oxygen concentrations are detectable only at high flowrates (> 50 µL/min), while coverage with a glass slide led to increased oxygen levels, even at lower flowrates (> 30 µL/min). In contrast, a significantly lower depletion of oxygen was determined for the Noa81 chip, where differences between the inlet and the outlet occurred only at very slow flowrates (< 4 µL/min). (b) Oxygen concentrations measured at the outlet of a standard PDMS (red curve) or a Noa81 (black) meander chip during the cultivation of *E. coli* biofilms under flow conditions. The chips were kept under oxic environmental conditions at room temperature and constantly perfused with air-saturated medium (20 µL/min, LB medium). As expected, the growth of *E. coli* biofilms led to consumption of oxygen, which was exhausted (< 0.02 % oxygen) after 22 h or 35 h of cultivation in the Noa81 or standard PDMS chip, respectively. Examination of the chips after 40 h with optical microscopy revealed only few cells in the Noa81 chip (d), whereas a thick biofilm layer was visible in the PDMS chip (c).

<u>Experimental details and discussion</u>

Possible strategies to reduce the gas permeability of the cultivation chip were evaluated by abiotic oxygen monitoring experiments described above. The PDMS body was bonded to cover slips from both sides, the top and the bottom of the demolded block (double sealed meander flowcell). And secondly, flowcells were fabricated in the commercially available thiolen polymer Noa81. This material was chosen because it offers several advantages like biocompatibility[2,3], transparency, low price and, most importantly, low gas permeability.[2] Furthermore, there are techniques available for the bonding of Noa81 structures to glass slides.[3]

To characterize the oxygen content of the medium inside the channel when enclosed in an anoxic environment, abiotic experiments with online oxygen measurement were conducted. To this end, the

various meander flowcells were placed in the anaerobic chamber which was constantly perfused with N_2 gas (Figure S1 h) and oxygen saturated medium was pumped through the channel. The oxygen content in the inlet and outlet chamber of the channel was measured online by optical sensors (Needle type micro sensor, OXY-10 Transmitter, Presens, Germany) and thereby the depletion of oxygen through the chip could be measured (Figure S1 e, f, h). The oxygen sensors were calibrated by a two-point method using oxygen saturated medium as 100 % and saturated sodium dithionate solution as 0 % reference. In Figure S2a the oxygen concentrations at the inlet and outlet chamber of the meander channels in the three mentioned variations are plotted after a sufficient equilibration period at various flowrates.

To evaluate whether a self-induced oxygen gradient can be achieved in the meander flowcells, despite the high gas permeability of the PDMS, a cultivation experiment was conducted. For comparison, *E. coli* biofilms were cultivated in PDMS as well as in a Noa81 meander chips. The oxygen content was monitored at the outlet throughout the whole cultivation. Figure S2 b shows representative curves of the oxygen content in dependency of the cultivation time at the outlet of a Noa81 and a PDMS flowcell during a cultivation experiment with *E. coli* under flow (20 µL/min, LB medium). The Chip was kept under oxic conditions at room temperature. The resulting biofilms inside the cultivation channel were examined by light microscopy. As shown in Figure S2 c, the PDMS cultivation channel was densely populated, whereas only few cells were visible inside the Noa81 channel (Figure S2 d).

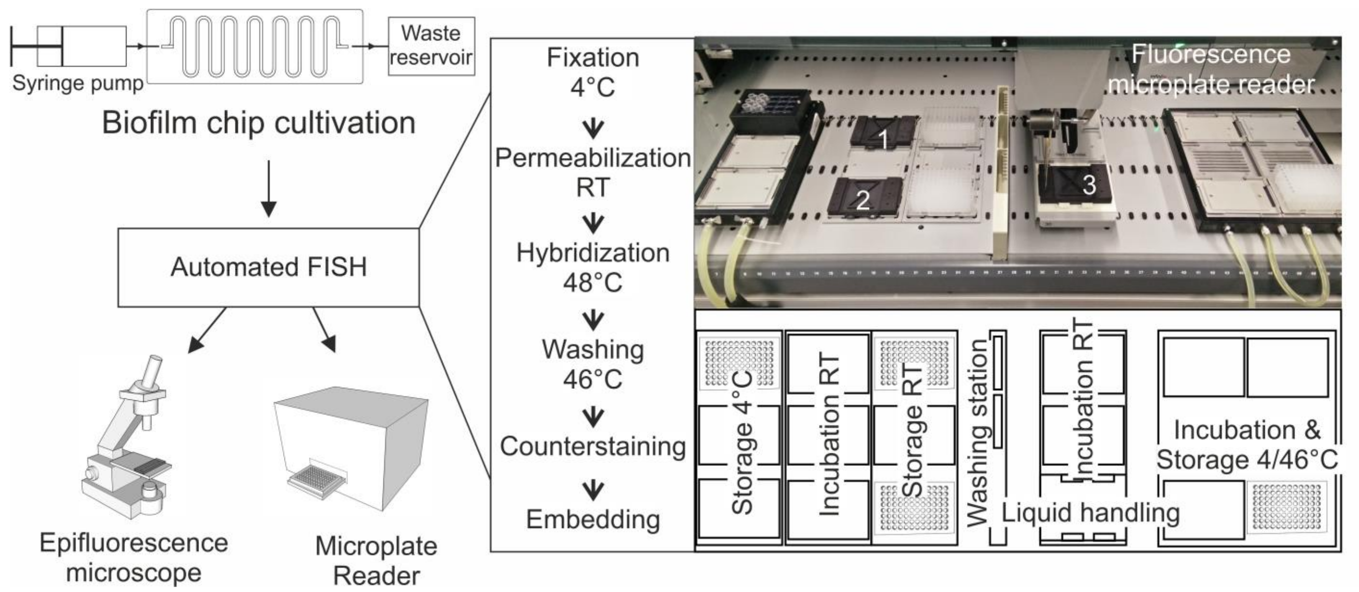

Supplementary Figure S3: Hardware for robotic end-point analysis of flow cell-cultivated biofilms. Biofilms are grown in the PDMS/glass hybrid cultivation flowcells. The various steps of FISH/CARD-FISH procedures are carried out fully automated with up to nine flowcells in parallel on a commercial liquid handling station (LHS). The LHS is illustrated by the photograph and the schematic representation of the working deck equipped with the various stations necessary for the FISH procedure. The fluorescence signals of FISH-labeled cells inside the flowcell's channel were determined with the on-deck microplate reader and subsequent analysis by epifluorescence microscopy. The system can handle nine flowcells contained in three cartridges (1, 2, 3) in parallel.

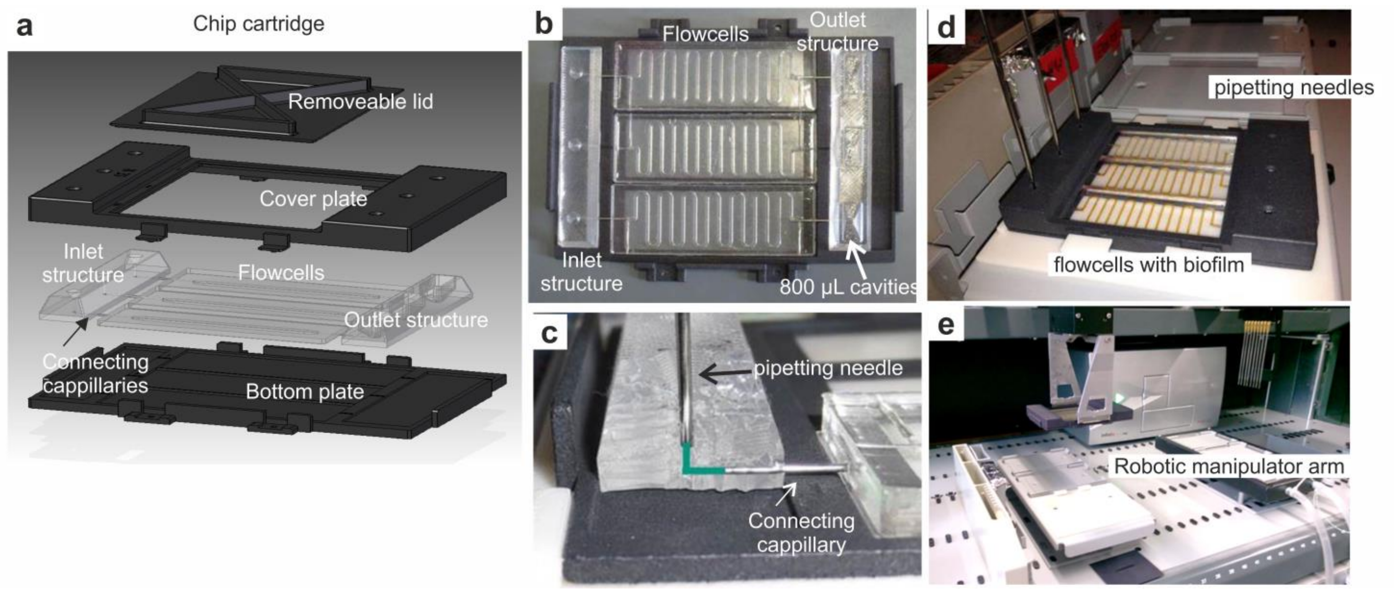

Supplementary Figure S4: Detailed description of the chip-to-LHS interface. (a) Exploded view of the newly developed chip-to-LHS interface. One cartridge can hold up to three flowchips which are connected to the inlet and outlet structures via stainless steel capillaries. (b) Photograph of the assembled parts of the chip-cartridge systems (without cover plate and lid). (c) Photograph of a cut open injection structure positioned on the chip cartridge's bottom plate. The pipetting needle of the LHS is inserted into the connector port and lowered to form a tight but reversible connection to the inlet structure. The capillary connects the outlet of the injection structure and the inlet of the microfluidic chip. The direction of the flow inside of the injection structure is marked in green color. (d) Overview of the pipetting needles of the LHS connected to the injection structure during the FISH staining of biofilms grown in the meander chip. (e) The cartridge designed for Auto FISH can be lifted and moved around by the robotic manipulator arm of the LHS.

Experimental details and discussion

The chip-to-LHS interface consisted of the flowcells, the inlet and outlet structures, connecting cannulas and the three-part cartridge. One cartridge can hold up to three cultivation chips for automated handling on the LHS (Figure S4). The PDMS structures for inlet and outlet were produced by solution casting of PDMS. The replication masters for this process as well as the flowcell cartridges were produced by 3D-printing (Sculpteo, France). The pipetting needles of the LHS could enter in the cone shaped connector ports of the inlet structure to establish the leak-free but reversible connection between the pipetting needles and the fluidic chip (Figure S4 c, d). The design of the PDMS inlet structure was adopted from the "microfluidics-on-liquid handling station" (µF-on-LHS) concept introduced by Waldbaur *et al.*[4]. The outlet structure was designed suitable for storage of up to 800 µL liquid in each cavity (Figure S4 b). The accumulated liquid could be removed from the cavities by the pipetting needles. The fully assembled fluidic system (injection structure, up to three flowcells, outlet structure) was placed in the carrier to allow for automated transfer by the robotic manipulator arm (RoMa) of the LHS (Figure S4e). The cartridge system comprised of the bottom plate with positioning recesses, a cover with access holes for the pipetting needles and a removable lid to enable optical analysis (Figure S a). The cover provided light protection and prevented lifting of the fluidic system from the bottom plate.

For automated execution of the FISH protocol, the liquid handling station Tecan Freedom Evo 200 was equipped with two temperature control systems with a coupled microplate/eppendorf tube carrier and a custom-made modification for a microplate carrier, which prevented the cartridge from lifting, when the pipetting needles were retracted. One temperature control system was constantly cooled to 4 °C and used for storage of heat-sensitive reagents (hybridization buffer, DAPI solution), the second temperature control system was initially cooled to 4 °C for the fixation step of the FISH assay and afterwards heated to 48 °C for hybridization. Temperature adjustments and all other process steps were controlled by custom-made .exe files initiated by the Evoware software. The automated FISH procedure followed the protocol from Pernthaler *et al.*[5] All reagents were injected to the channels by the LHS with a flowrate of 50 µL/min.

The fluorescence of the *E. coli* & *B. subtilis* biofilms was measured with the on-deck microplate reader (Tecan, Switzerland). The detachable lid of the carrier system was opened by the RoMa and a predefined measurement protocol was executed automatically. A modified protocol was used for the imaging of 13 measuring points in each chip uniformly distributed along the flow path. The excitation wavelengths for detection of the probes were 490 nm and 556 nm for Alexa 488 and Alexa 546, respectively. The emission was determined at 530 nm and 588 nm for Alexa 488 and Alexa 546, respectively. The gain was set to 150 and 50 flashes were applied per measurement. DAPI was excited at 358 nm, emission was detected at 461 nm. For DAPI the gain was set to 80 and 25 flashes were used per measurement. For each individual chip, the background values for the 13 measurement points (Figure 3 b) were recorded beforehand and subtracted from the raw signal. The mean value of all 13 measurement points yielded the signal of one cultivation channel which was then normalized to the mean background value of the respective chip. The average signals and the standard deviation obtained in three in parallel cultivated chips were determined (Figure 3 c).

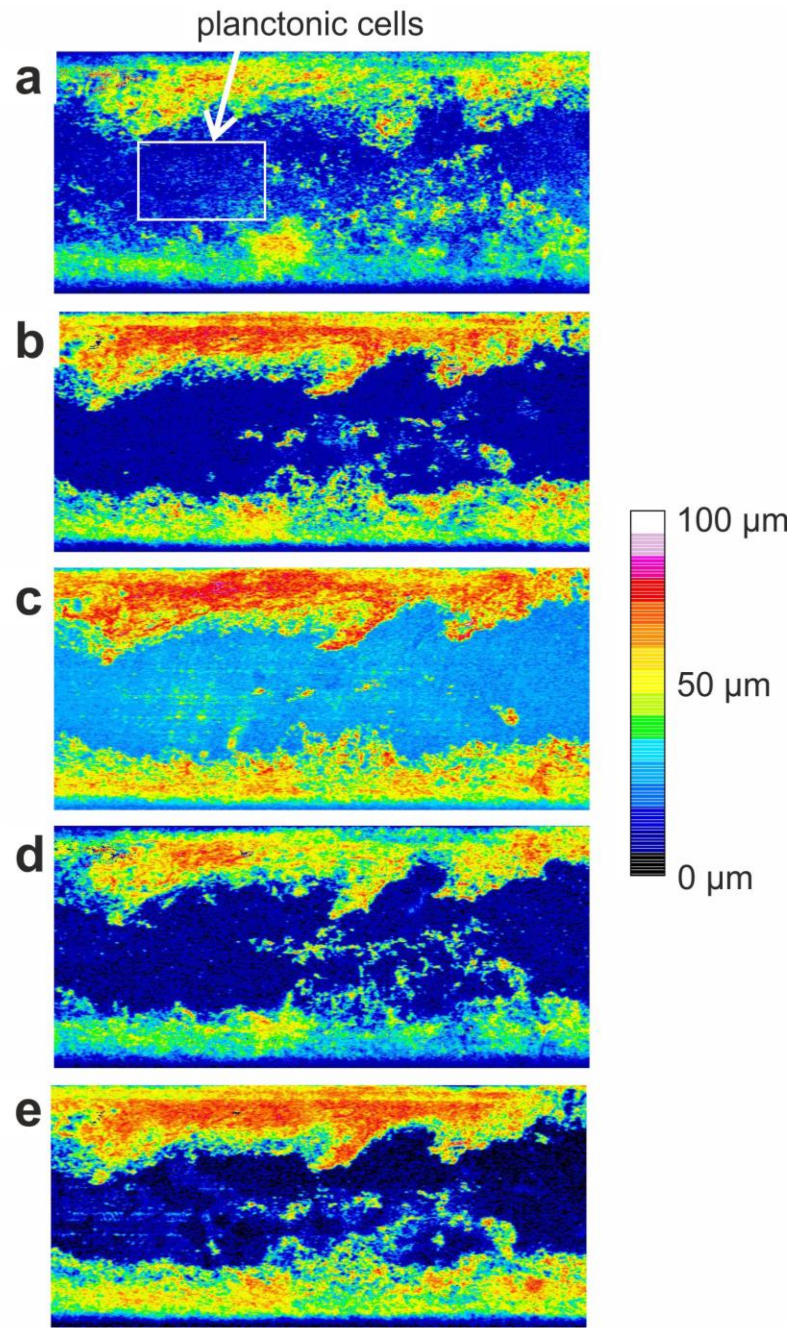

Supplementary Figure S5: OCT analysis of biofilms during FISH procedure. Topographic representation (6.7 × 3 mm², height 0-100 µm, as indicated by color scale) of the 3D structure of a mixed species *E. coli* and *B. subtilis* biofilm cultured in the straight cultivation chip obtained by OCT during the automated FISH procedure captured. To analyze the structural changes of the biofilm, which may occur during the various treatments of the automated FISH procedure, images were acquired immediately before FISH (a), after fixation (b), permeabilization (c), hybridization (d) and washing (e) steps. Although slight biofilm detachment was observed in selected regions, the overall structural integrity remained preserved throughout the entire procedure.

<u>Experimental details and discussion</u>

To prove the structural integrity of the biofilms during the automated FISH procedure, we monitored the mesoscopic structure of the developing mixed biofilms (*E. coli* & *B. subtilis*) by means of optical coherence tomography (OCT) during the entire experiment.

Briefly, OCT is an interferometric imaging modality capable of visualizing biofilms completely [6,7]. In this study a GANYMEDE-II spectral domain OCT was applied (Thorlabs GmbH, Dachau, Germany). It acquires three-dimensional structural datasets at the mesoscale (mm-range) with high lateral (≤ 12 µm/pixel) and axial (≤ 3.1 µm/pixel) resolution at high speed, in situ, and without any sample treatment directly inside the cultivation device. Thus, the biofilm structure after each step of the automated FISH procedure was acquired and stored in a three-dimensional dataset. These datasets were analysed using ImageJ.[8] After cropping the datasets to the flow channel of the microfluidic device and binarization using Otsu's method[9] a

topographic representation of the bulk-biofilm interface was calculated. Figure S5 shows the subsequent OCT images during automated FISH procedure as well as an example of an OCT image in conjunction to the FISH images itself. A lot of planktonic cells were visible before the protocol was started (green, lose parts), which were washed out in the fixation step. Subsequently, there was no obvious association between biomass loss and one specific step, rather than a continuous slight erosion of biofilm throughout the whole procedure. We observed partly strong reflexion effects on the glass bottom of the cultivation channel which prohibited the exact quantification of the attached biofilms.

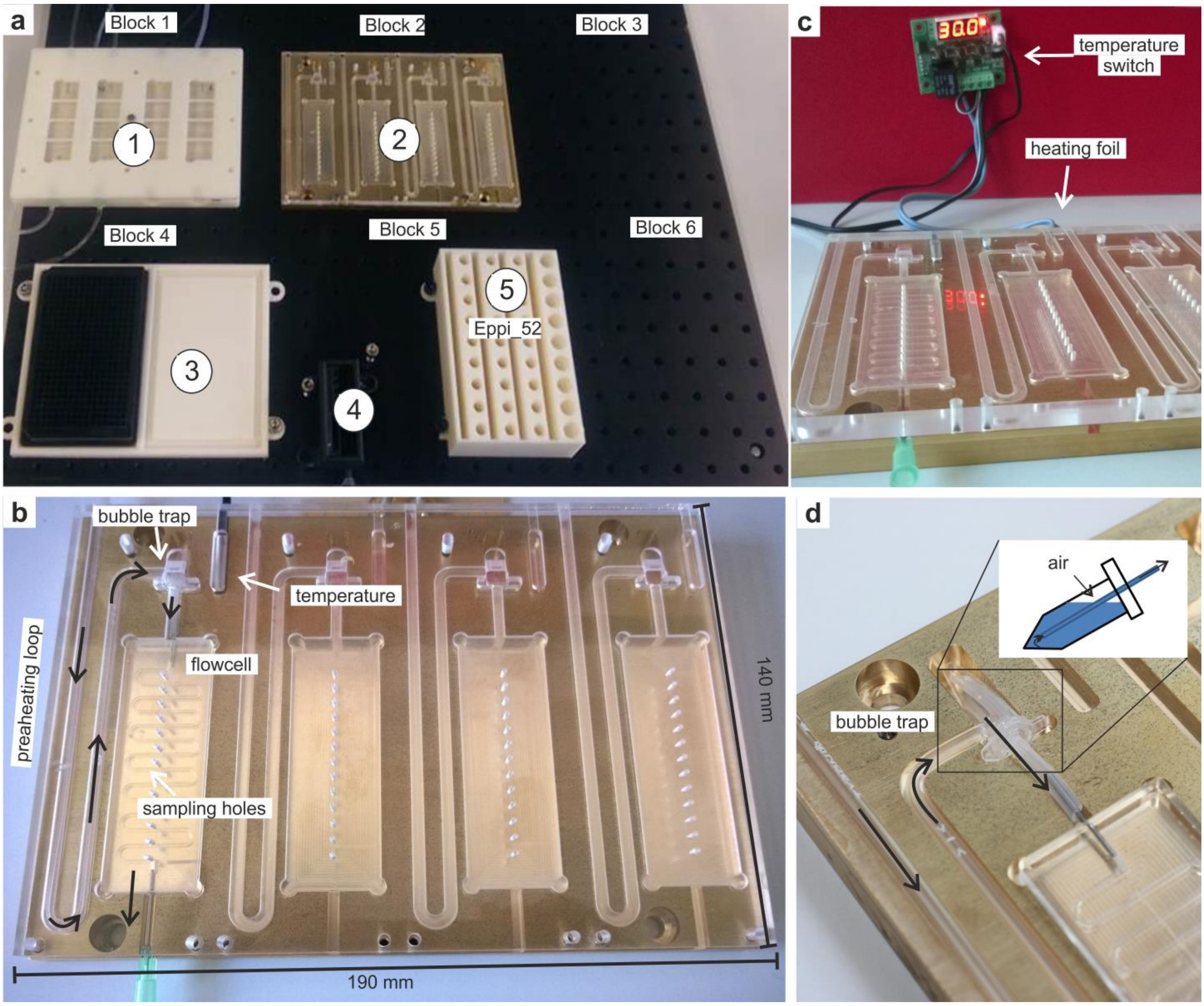

Supplementary Figure S6: Robotic deck of the sampling device. (a) The robotic deck is organized in blocks on an aluminum pegboard. Custom-made accessories, the temperature controlled chip holders (1, 2), the microplate holder (3), the washing station (4), and the reaction tube holder (5) are arranged at fixed positions on the deck. (b) Close-up of the cultivation chip holder for mounting up to four flowcells directly on a temperature-controlled heating foil (c). Each recess for the flowcell is connected to the medium supply through a 24 cm preheating loop and a bubble trap (d) to equilibrate medium temperature and remove air bubbles, respectively. The flow of medium is indicated by arrows in images b and d. The entire cultivation chip holder is capped with a transparent PMMA lid that contains holes for online sampling from the flowcells.

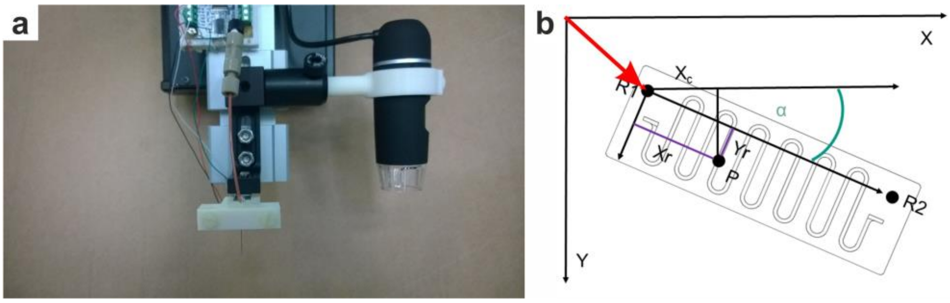

Supplementary Figure S7: Automatic chip recognition. (a) Photograph of the sampling arm with attached camera. (b) Schematic representation of the functioning of the camera recognition system of the developed robotic platform. Two reference positions on the chip that are directly incorporated in the replication master are recognized by the image analysis tool[10] and used for calculation of the coordinates of predefined sampling points of flowcell chips.

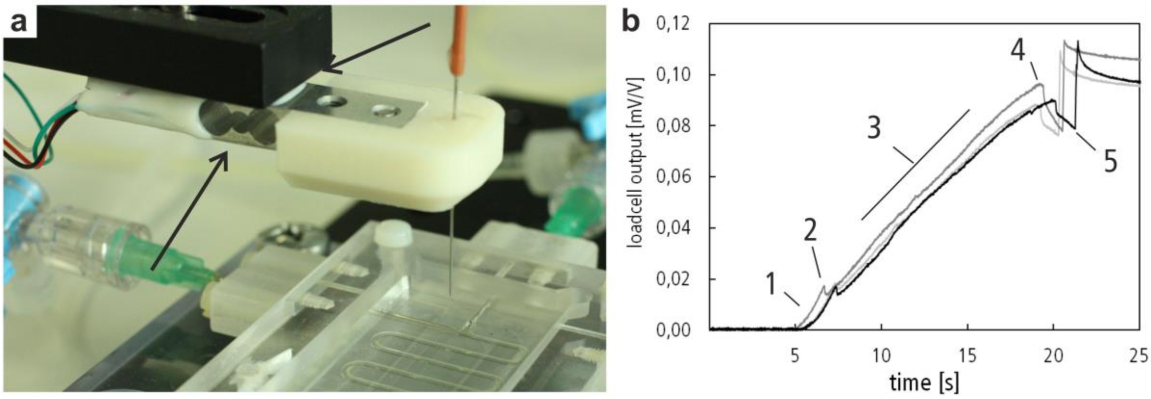

Supplementary Figure S8: Mounting of the probing cannula on a pressure-sensitive load cell. a) Close up view of the probing cannula, which is mounted on the pressure-sensitive loadcell. Arrows indicate the positions of the strain gauges, which measure the shear stress occurring in the metal body of the load cell. b) Data of the force value recorded by the load cell during piercing of the meander cultivation chip. In the first phase (1) the stress increases due to elastic deformation of the PDMS layer. A small first stress release occurred when the PDMS is ruptured (2). Subsequently, the detected force increases again due to friction (3). When the tip of the cannula enters the channel (4), the force value drops until the glass bottom of the cultivation channel is reached (5), thus leading to a sudden increase in the detected stress.

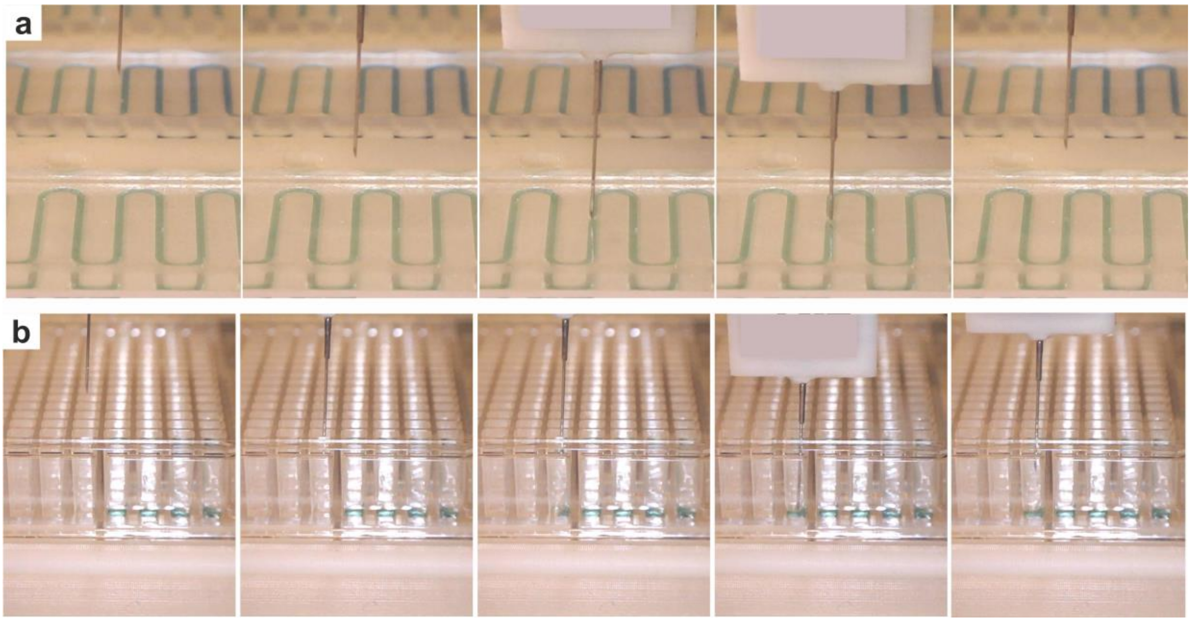

Supplementary Figure S9: Image series of the probing cannula entering the cultivation channel (a) and depositing 10 µL samples into wells of a 384 well microplate plate (b).

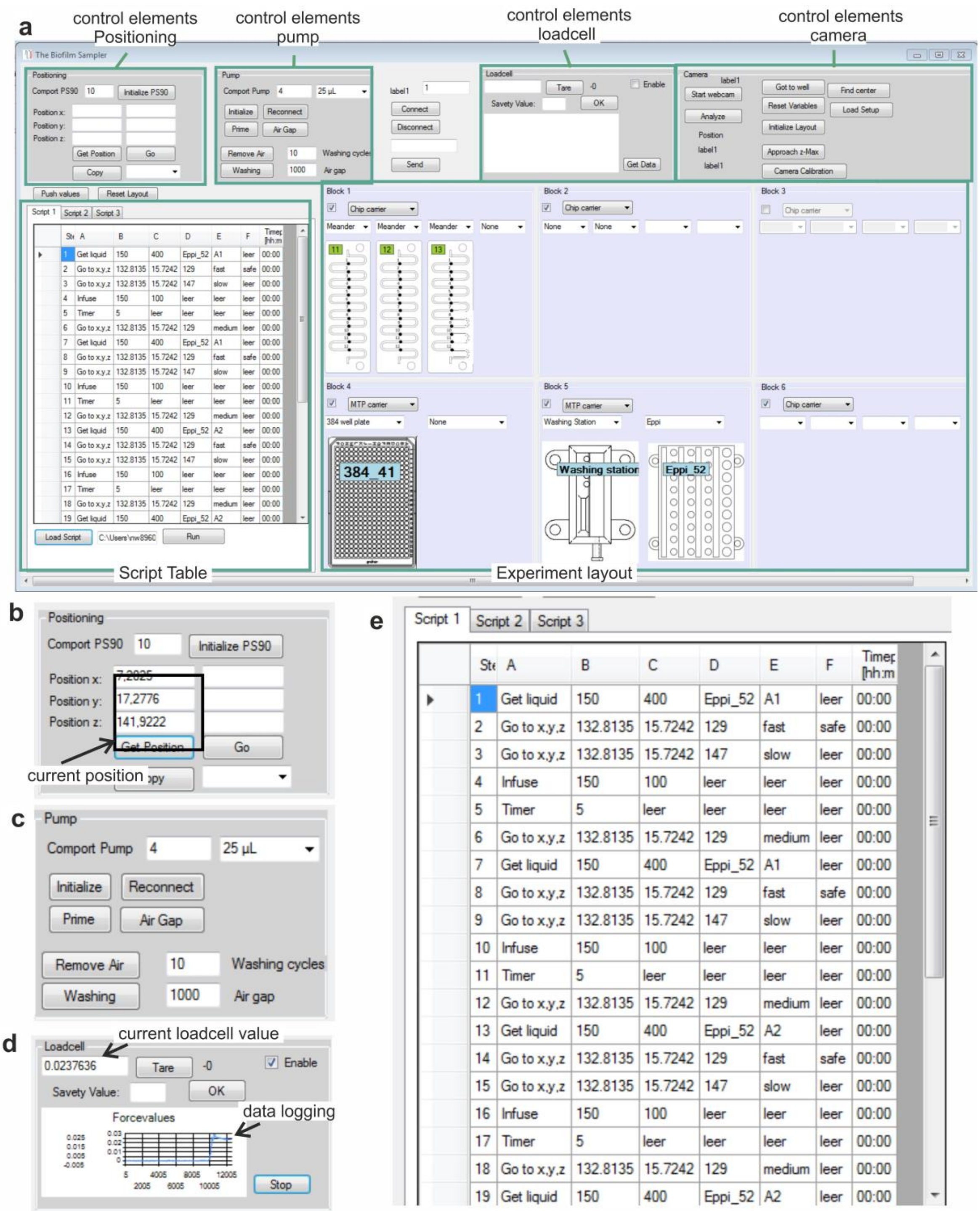

Supplementary Figure S10: Graphical user interface (GUI) for the control of the biofilm sampler. The software was realized in Visual C#, an object-oriented programming language. (a) The GUI is divided into direct and script control elements. In the upper part, various elements allow the direct manipulation of the different components, i.e., positioning stages, the pumping unit, or the camera installed on the sampling probe (Figure S7). The lower part contains the elements necessary to control the platform. Every usable lab ware is assigned to a defined ID in the dynamic representation of the robotic deck, which is also organized in six blocks. For each lab ware, the corresponding position data is stored and can be retrieved under the respective ID. Depending on the experiment, the user can freely choose between a chip holder, a microplate holder and two reaction tube holders. The images b) - e) show detailed views of the control elements for the positioning system (b), the pump (c), the loadcell (d) and the script table (e). The commonly used spreadsheet program Excel can be used to create script files for control of the biofilm sampler. Prescribed script modules can be used to enable various procedures. The modules can be customized by numerous freely definable variables, such as volume and flowrate, directly in the Excel template. The finished script is then imported to the script table (e).

References

1 Hung, L. H., Lin, R. & Lee, A. P. Rapid microfabrication of solvent-resistant biocompatible microfluidic devices. *Lab Chip* **8**, 983-987, doi:10.1039/b717710k (2008).
2 Hung, L.-H., Lin, R. & Lee, A. P. Rapid microfabrication of solvent-resistant biocompatible microfluidic devices. *Lab Chip* **8**, 983-987 (2008).
3 Morel, M., Bartolo, D., Galas, J.-C., Dahan, M. & Studer, V. Microfluidic stickers for cell-and tissue-based assays in microchannels. *Lab Chip* **9**, 1011-1013 (2009).
4 Waldbaur, A., Kittelmann, J., Radtke, C. P., Hubbuch, J. & Rapp, B. E. Microfluidics on liquid handling stations (μF-on-LHS): an industry compatible chip interface between microfluidics and automated liquid handling stations. *Lab Chip* **13**, 2337-2343 (2013).
5 Pernthaler, A. *et al.* Sensitive multi-color fluorescence *in situ* hybridization for the identification of environmental microorganisms. *MMEM-3.11*, 711-725 (2004).
6 Haisch, C. & Niessner, R. Visualisation of transient processes in biofilms by optical coherence tomography. *Water Res.* **41**, 2467-2472 (2007).
7 Wagner, M., Taherzadeh, D., Haisch, C. & Horn, H. Investigation of the mesoscale structure and volumetric features of biofilms using optical coherence tomography. *Biotechnol. Bioeng.* **107**, 844-853 (2010).
8 Schindelin, J. *et al.* Fiji: an open-source platform for biological-image analysis. *Nat. Methods* **9**, 676-682 (2012).
9 Otsu, N. An automatic threshold selection method based on discriminate and least squares criteria. *Trans. Electron. Commun. Eng. Jpn. D J.* **63**, 349-356 (1980).
10 Kirillov, A. *AForge.NET Framework*, <http://www.aforgenet.com/> (2011).
